# Nanoemulsion Based Vehicle for Effective Ocular Delivery of Moxifloxacin Using Experimental Design and Pharmacokinetic Study in Rabbits

**DOI:** 10.3390/pharmaceutics11050230

**Published:** 2019-05-11

**Authors:** Jigar Shah, Anroop B. Nair, Shery Jacob, Rakesh K. Patel, Hiral Shah, Tamer M. Shehata, Mohamed Aly Morsy

**Affiliations:** 1 Department of Pharmaceutics, Institute of Pharmacy, Nirma University, Ahmedabad, Gujarat 382481, India; jigsh12@gmail.com; 2 Department of Pharmaceutical Sciences, College of Clinical Pharmacy, King Faisal University, Al-Ahsa 31982, Saudi Arabia; momorsy@kfu.edu.sa; 3 Department of Pharmaceutical Sciences, College of Pharmacy, Gulf Medical University, Ajman 4184, UAE; sheryjacob6876@gmail.com; 4 Shree S.K. Patel College of Pharmaceutical Education and Research, Kherva, Ganpat Vidyanagar, Mehsana, Gujarat 384012, India; rakesh.patel@ganpatuniversity.ac.in; 5 Arihant School of Pharmacy & BRI, Gandhinagar, Gujarat 382421, India; jigshir123@gmail.com; 6 Department of Pharmaceutics and Industrial Pharmacy, Faculty of Pharmacy, University of Zagazig, Zagazig 44519, Egypt; tamershehata@zu.edu.eg; 7 Department of Pharmacology, Faculty of Medicine, Minia University, El-Minia 61511, Egypt

**Keywords:** nanoemulsion, mixture design, aqueous humor, antimicrobial activity

## Abstract

Nanoemulsion is one of the potential drug delivery strategies used in topical ocular therapy. The purpose of this study was to design and optimize a nanoemulsion-based system to improve therapeutic efficacy of moxifloxacin in ophthalmic delivery. Moxifloxacin nanoemulsions were prepared by testing their solubility in oil, surfactants, and cosurfactants. A pseudoternary phase diagram was constructed by titration technique and nanoemulsions were obtained with four component mixtures of Tween 80, Soluphor^®^ P, ethyl oleate and water. An experiment with simplex lattice design was conducted to assess the influence of formulation parameters in seven nanoemulsion formulations (MM1–MM7) containing moxifloxacin. Physicochemical characteristics and in vitro release of MM1–MM7 were examined and optimized formulation (MM3) was further evaluated for ex vivo permeation, antimicrobial activity, ocular irritation and stability. Drug pharmacokinetics in rabbit aqueous humor was assessed for MM3 and compared with conventional commercial eye drop formulation (control). MM3 exhibited complete drug release in 3 h by Higuchi diffusion controlled mechanism. Corneal steady state flux of MM3 (~32.01 µg/cm^2^/h) and control (~31.53 µg/cm^2^/h) were comparable. Ocular irritation study indicated good tolerance of MM3 and its safety for ophthalmic use. No significant changes were observed in the physicochemical properties of MM3 when stored in the refrigerator for 3 months. The greater aqueous humor concentration (*C*_max_; 555.73 ± 133.34 ng/mL) and delayed *T*_max_ value (2 h) observed in MM3 suggest a reduced dosing frequency and increased therapeutic efficacy relative to control. The area under the aqueous humor concentration versus time curve (*AUC*_0–8 h_) of MM3 (1859.76 ± 424.51 ng·h/mL) was ~2 fold higher (*p* < 0.0005) than the control, suggesting a significant improvement in aqueous humor bioavailability. Our findings suggest that optimized nanoemulsion (MM3) enhanced the therapeutic effect of moxifloxacin and can therefore be used as a safe and effective delivery vehicle for ophthalmic therapy.

## 1. Introduction

The ocular drug delivery system is one of the most attractive and challenging drug delivery systems for pharmaceutical scientists [1]. Conventional eye drops account for more than 90% of the available ophthalmic formulations but the efficiency of these products is limited by transient residence time, low permeability of corneal epithelium, rapid pre-corneal loss and high tear fluid turnover [2]. However, less than 5% of the drugs contained in eye drops penetrates the corneal membrane and reaches the intraocular tissues; and the remaining dose usually undergoes trans-conjunctival absorption or trans-nasal absorption or drainage via the nasolacrimal duct. Consequently, extensive research has been conducted to improve the effectiveness of topical ocular therapy by developing drug delivery systems which can increase ocular retention, improve trans-corneal drug absorption and reduce systemic adverse effects while retaining the ease of application and benefit of eye drops. Various drug delivery strategies such as bioadhesive hydrogels, temperature or pH-sensitive in situ gel forming systems, collagen shields, colloidal dosage forms like microparticles, microemulsions, nanoparticles, nanosuspensions, nanoemulsions, liposomes, niosomes, nanomicelles, and dendrimers have been formulated and evaluated to partially or fully achieve these objectives [3]. Among various delivery approaches, the colloidal systems received greater attention due to their potential to improve corneal penetration, greater retention at ocular surface, as well as ease of administration similar to eye drop solutions [4]. Despite the fact that the colloidal ocular drug delivery systems like liposomes and niosomes offer certain improvement over conventional liquid dosage forms, the major limitations which have limited their prospects include their inherent tendency to aggregate, the instability and leakage of entrapped drug have limited their future prospect [5,6]. 

Nanoemulsions are thermodynamically stable and optically transparent fine dispersion (10–200 nm) of multi-component fluids, and frequently consist of an aqueous phase, an oily phase, a primary surfactant as an emulsifying agent, a cosurfactant generally an alkanol of intermediate chain length and occasionally an electrolyte [7]. The major benefits of this colloidal dispersion include enhancement in ocular retention and extended duration, reduced drug protein binding, improved corneal drug permeation, sustained drug release, reduced systemic adverse effects and ease of use for the incorporation of both hydrophilic and lipophilic drugs. In addition, nanoemulsions can interact with the lipid layer of the tear film, can stay in the conjunctival sac for longer times, and subsequently act as a drug depot [8]. The potential of nanoemulsions as a promising alternative for conventional eye formulation in treating various ocular diseases of both the anterior and posterior ocular segments has been described in literature [4]. In this context, moxifloxacin, a fourth-generation fluoroquinolone antibiotic is commercially available as an ophthalmic solution (0.5% *w*/*v*) and is used for the treatment of bacterial conjunctivitis or other bacterial infections of the eyes. Moxifloxacin acts by inhibiting the DNA gyrase and topoisomerase IV required for bacterial DNA replication, repair, and recombination. It was reported that moxifloxacin has enhanced efficacy, safety and tolerance in comparison to older fluoroquinolone derivatives [9]. However, the conventional therapy of this drug in ocular therapy is limited by short residence time. Hence, encapsulating moxifloxacin in droplets that form a nanoemulsion could be an alternative for its ophthalmic use. The objective of this investigation was to optimize the moxifloxacin-loaded nanoemulsion system, characterize and compare the in vivo ocular efficacy with the commercial eye drop. A pseudoternary phase diagram of four component mixtures was constructed by titration technique. Selected nanoemulsions were characterized for pH, droplets size, polydispersity index, zeta potential, conductivity, viscosity, drug content, and dilution potential. The optimized nanoemulsion (MM3) was further evaluated for corneal permeation, antimicrobial effect, ocular irritation and in vivo drug pharmacokinetics in the aqueous humor of rabbits. 

## 2. Materials and Methods

### 2.1. Materials

Moxifloxacin (molecular weight of 437.9 Da) was received as an in-kind gift from Zydus Cadila Ltd., Ahmedabad, India with a purity of 99.99%. Soluphor^®^ P was generously supplied by BASF, Ludwigshafen, Germany. Tween 80 was purchased from S.D Fine Chem, Mumbai, India and ethyl oleate was purchased from Central Drug House Pvt. Ltd., Mumbai, India. All other chemicals used were of analytical grade.

### 2.2. Drug Analysis

The amount of moxifloxacin was determined by the high-performance liquid chromatography (HPLC) method described in the literature [10]. A HPLC system (1525, Waters, Milford, MA, USA) consisting of a Discovery C18 column (250 mm × 4.6 mm, i.d, 5 μm) was used. Chromatographic separation was carried out using a mixture of methanol: acetonitrile: water (85:5:10, *v*/*v*/*v*), pH 2.75 adjusted with phosphoric acid was delivered at a flow rate of 1 mL min^−1^ with an injection volume of 25 µL. The isocratic elution was done at 25 °C and monitored using UV detector at 290 nm.

### 2.3. Development of Pseudoternary Phase Diagram

The pseudoternary phase diagram of four component mixtures of oil (ethyl oleate), surfactant (Tween 80), cosurfactant (Soluphor P) and water was constructed by titration technique to obtain concentration ranges that can result in large existence with the nanoemulsion region at room temperature [11]. A titration technique was employed for the preparation of the pseudoternary phase diagrams [12]. Briefly, Tween 80 was blended with Soluphor P in a fixed weight ratio of 1:1, 1:1.5, 1:2, 1:2.5, 1:3, 1:4 and 1:5, respectively. Aliquots of each Tween 80 and cosurfactant mixture (*S*_mix_) were then mixed with oil phase at room temperature (25 °C). The ratio of oil to *S*_mix_ was varied as 9:1, 8:2, 7:3, 6:4, 5:5, 4:6, 7:3, 2:8 and 1:9. Then, water was added to the above mixture in 5% increments and checked for formation of emulsion or liquid crystal or gel. The phases were identified using visual inspection, microscopic inspection, and measurement of droplets size. The resulting nanoemulsion was tightly sealed and stored at ambient temperature. The points corresponding to mixture components that resulted in nanoemulsion were noted and marked in a phase diagram region. The resulted phase diagrams of different surfactant: Cosurfactant ratios were compared and the ratio that could result in the large existence area of nanoemulsion domain in phase diagram was selected for further optimization.

### 2.4. Preparation of Moxifloxacin Nanoemulsion

Moxifloxacin nanoemulsions (o/w) were formulated by appropriate quantity of deionized water, ethyl oleate, Tween 80 and Soluphor P. Required amount of moxifloxacin (0.5%, *w*/*v*) was dissolved in ethyl oleate. To *S*_mix_, an adequate amount of ethyl oleate was added and mixed. Then the mixture was titrated by gradual addition and mixing of distilled water in order to achieve the equilibrium immediately. Further, it was thoroughly stirred and vortexed to obtain nanoemulsion. To maintain sterility of the prepared formulation, benzalkonium chloride (0.005%, *w*/*w*) was used as a preservative and stored in multi-dose containers [13].

### 2.5. Experimental Design

**Figure 1:**
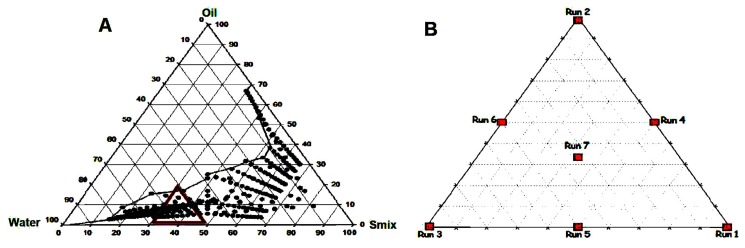
Pseudo ternary phase diagram showing nanoemulsion region (**A**) and distribution for each of run in a mixture design (**B**).

The objective of modeling the phase diagrams is to quantify the effect of formulation composition on the droplets size. A successful “mixture design” shows the statistical approach to obtain the relationship between the droplets size distribution and the amounts of various components. In this method, the pseudoternary phase diagrams were plotted and several points were selected within the nanoemulsion region for particle size measurement. The points (black dots) represent the nanoemulsion region in Figure 1A. Within the nanoemulsion region, a triangular area was arbitrarily selected and marked with a red triangle (Figure 1A) which shows different runs of mixture design and the enlarged version is represented in Figure 1B. Hence, Figure 1B shows the sketch diagram of Run (trial) 1 to Run 7 of mixture design. The constraint that the proportions of different components must sum to 100% should be satisfied. According to Figure 1B, the points can be chosen such as three vertexes, three halfway points between vertices and the center point. Each vertex represents a formulation containing the maximum quantity of one component, with the other two components at a minimum amount. The halfway point between the two vertices illustrates a formulation incorporating the average of the minimum and maximum quantity of the two constituents represented by the respective apex. The centre point shows a formulation containing one third of the individual component. A total of seven formulations (MM1–MM7) were opted from the nanoemulsion region for further study and their compositions are summarized in Table 1. 

### 2.6. Characterization of Moxifloxacin Nanoemulsions 

#### 2.6.1. Drug Content and pH

Drug content in the prepared nanoemulsions (MM1–MM7) was determined using HPLC. The pH of formulations (MM1–MM7) were measured by a calibrated pH meter (Mettler Toledo MP-220, Greifensee, Switzerland). 

#### 2.6.2. Transmittance, Conductivity and Dilution Potential

The percentage of the transmittance of nanoemulsions was measured using colorimeter (Photoelectric Colorimeter 113, Systronics, Ahmedabad, India). Percentage transmission was set to zero using filter and 100% using transparent cuvette filled with water. Then, different nanoemulsion samples were kept in the transparent cuvette and percentage transmission was measured. Electrical conductivity of nanoemulsions was studied using a conductometer to determine the type. Briefly, an electrode was totally immersed and fixed in the nanoemulsion (20 mL) and the temperature was raised to 1 °C/min steadily. The nanoemulsion was agitated with a stirrer, and the change in the conductivity was recorded. To determine dilution potential, the nanoemulsion was diluted 10 times with continuous media and the occurrence of phase separation was noted.

#### 2.6.3. Particle Size Characterization and Zeta Potential

The droplets size, size distribution and polydispersity index of nanoemulsions were analyzed employing a dynamic light scattering technique using Malvern Zetasizer (Nano ZS90, Malvern Instruments, Malvern, UK) at 25 °C. In brief, a few drops of respective samples were added directly to a polystyrene disposable cuvette and fixed in the direction of laser light beam. The scattered light signal was measured with a detector placed at a right angle and the droplets size were determined based on the physical properties of the scattered light such as the angular distribution, frequency shift, the polarization and the intensity of the light [14]. For the zeta potential measurement, samples were diluted with deionized water and the electrophoretic mobility values was determined at 25 °C using the software DTS, version 4.1 (Malvern, England, UK, 2009). 

#### 2.6.4. Viscosity 

Viscosity of nanoemulsions was measured at different angular velocities at a temperature of 25 °C using the Brookfield synchro-Lectric viscometer (LVDVI prime, Middleborough, MA, USA). 

### 2.7. Transmission Electron Microscopy (TEM)

Structural morphology of nanoemulsion droplets was investigated with TEM (Tecnai 20, Philips, Holland, OR, USA) operated at 200 kV and of a 0.15 nm efficient in comprehensive resolution. The bright field imaging technique with high magnification and diffraction modes was used to examine the morphology and structure of the nanoemulsion globules [15]. TEM imaging was carried out by staining a drop of the nanoemulsion with phosphotungstic acid solution (2% *w*/*v*) and directly placing on the copper grids, subsequently dried at room temperature (25 ± 2 °C).

### 2.8. In Vitro Release

The drug release studies were performed employing a vertical Franz diffusion cell having an effective surface area of 1.13 cm^2^ and simulated tear fluid (pH 7.4) as the dissolution medium [16]. Briefly, 1 mL of nanoemulsion (equivalent to 5 mg of moxifloxacin per ml) or drug solution (control) in the stimulated tear fluid was taken on a previously soaked cellophane dialyzing membrane (MWCO 12–14 kDa, Spectra/por^®^ Spectrum Laboratories Inc., Rancho Dominguez, Berkeley, CA, USA) that separates the donor and receptor cell. The entire assembly was kept on a thermostatically controlled water bath set at 37 ± 0.5 °C and receptor medium was stirred at 50 rpm. Aliquots of sample (1 mL) were drawn at regular time intervals (0.5, 1, 2, 3, 4, 5 and 6 h) and replaced with the same volume of fresh media. The samples were subsequently diluted and analyzed for moxifloxacin content by HPLC. The data were analyzed to determine correlation coefficient (*r*^2^) and release kinetics using various mathematical models [17];

- Zero order model *Q* = *Q*_0_ + *kt*
- First order model *Q* = *Q*_0_ × e*^kt^*
- Higuchi model *Q* = *k* × *t*^0.5^
- Hixson-Crowell model *Q*^1/3^ = *kt* + *Q*_0_^1/3^
- Korsmeyer–Peppas model *Q* = *k* × *t^n^*
- Weibull model *Q* = 1 − exp[−(*t*)*^b^*^/*a*^]

where *Q* represents quantity of drug released in time *t*, *Q*_0_ represents value of *Q* at zero time, *k* represents the rate constant, *n* represents the diffusional exponent, *a* represents the time constant and *b* represents the shape parameter. The correlation coefficient (*r*^2^) and the order of release pattern was calculated in each case. 

### 2.9. Ex Vivo Permeation 

The permeation studies were carried out using the Franz diffusion cell with a standard setup previously used in our earlier study [18]. Briefly, optimized formulation (MM3) or control (commercial eye drops; Vigamox™) (equivalent to 5 mg of moxifloxacin per mL) was placed in donor compartment and simulated tear fluid (pH 7.4) in the receptor compartment. Between donor and receptor compartment, the isolated rabbit cornea membrane was placed. The temperature of the medium was maintained at 37 ± 0.5 °C by the circulation of warm water through the outer jacket. Samples were withdrawn at predetermined time intervals (up to 6 h) and the same volume of fresh medium was replaced. The withdrawn samples were diluted and analyzed by HPLC.

### 2.10. Antimicrobial Efficacy

The antimicrobial efficacy of MM3 was determined by the agar diffusion test employing the cup plate technique and compared with control (commercial eye drops of moxifloxacin). The sterile products (MM3 or control) were poured (0.01 mL) into the cups of sterile nutrient agar previously inoculated with susceptible test organisms (*Pseudomonas aeruginosa* and *Staphylococcus aureus*) under horizontal laminar air flow hood. After allowing diffusion of the solutions for 2 h, the agar plates were incubated at 37 °C for 24 h. The zone of inhibition measured around each cup was compared with the control. Both positive (with test organisms) and negative controls (without test organisms) were maintained throughout the study.

### 2.11. Ocular Irritation 

Albino rabbits (2–3 kg) placed in an animal house under observation were given ad libitum access to water and diet for 24 h. The guidelines of the Institutional Animal Ethics Committee were strictly followed while performing experiments (IPS/PCEU/PHD10/002). In vivo ocular irritation studies were performed according to the Draize technique [19]. Single administration of 60 µL was instilled in the left eye of each rabbit while keeping the untreated eye as the control. The sterile MM3 was administered twice daily for a period of 21 days (1, 2, 3, 4, 7, 10, 15, 18 and 21 days). The rabbits were observed periodically for redness, swelling and watering of the eye. 

### 2.12. Pharmacokinetics 

In vivo pharmacokinetic studies were carried out in Albino rabbits (2–3 kg). Animals were separated into two groups of each containing six rabbits. All animal procedures were performed at the Nirma University following the guidelines (IPS/PCEU/PHD10/002, dated; 23/01/2010, Scientific Research Ethics Committee, Nirma University). Rabbits were treated with MM3 or control (commercial eye drops). For the first group, drops (50 μL of 0.5% *w*/*v* moxifloxacin) of MM3 was instilled in the lower cul-de-sac of both eyes of each rabbit [20], and the upper and lower eyelids were gently held closed for 2 min to maximize drug cornea contact. In a similar manner, both eyes of each rabbit of the second group were given single topical instillation (50 μL of 0.5% *w*/*v* moxifloxacin) of commercial eye drops. Each rabbit was anaesthetized by intra muscular injection of ketamine (50 mg/kg) and xylazine (10 mg/kg) [21]. Additionally, lidocaine (2% *w*/*v*) was applied at the injection site to provide local anesthesia. Then eyelash and eye liners/lids were wiped with povidone solution (5% *w*/*v*) to maintain standard of care to give intra-ocular injection. A 29-gauge insulin syringe needle was used to collect aqueous humor (0.5, 1, 2, 4 and 8 h) and samples (50 μL) were mixed with acetonitrile, and stored at −80 °C. The mixture was then centrifuged at 3000 rpm for 15 min and the supernatant was analyzed for drug content using HPLC. 

### 2.13. Stability 

Sterile nanoemulsion (MM3; 5 mL) was stored in an amber colored container for a period of three months in the refrigerator (2–8 °C). Stability of MM3 was routinely evaluated by visual inspection of the samples initially on a daily and later on a weekly basis for pH, phase separation, flocculation or precipitation [22]. Stability of nanoemulsion was also checked by centrifugation (Remi Centrifuge, Mumbai, India) by 12,000 rpm for 30 min and then the clarity, phase separation and concentration of drug were investigated. Droplet size of the nanoemulsion upon storage was also determined to assess the stability in terms of drastic changes in the mean droplet diameter due to droplet coalescence or aggregation.

### 2.14. Data Analysis

A plot of the cumulative amount of moxifloxacin permeated across the cornea was plotted against time and the slope was measured as flux [23]. The statistical evaluation of the data was analyzed using one-way analysis of variance (ANOVA) (SPSS 23, SPSS Inc., Chicago, IL, USA). The statistical differences between values showing *p* < 0.05 were considered as significant.

## 3. Results and Discussion

### 3.1. Pseudoternary Phase Diagram

Preliminary studies were carried out to select ingredients for preparing moxifloxacin nanoemulsion. Indeed, the selection of the vehicle is extremely important as it provides good solubilizing efficiency of the drug, which is essential for constituting a nanoemulsion [24]. Hence, the solubility of moxifloxacin in various oils, surfactants, and cosurfactants was determined by the standard procedures. Based on the high moxifloxacin solubility, ethyl oleate (oil; solubility ~28.37 mg/mL), Tween 80 (surfactant; solubility ~10.56 mg/mL) and Soluphor P (cosurfactant; solubility ~7.22 mg/mL) were selected for nanoemulsion preparation. The utility of the selected vehicles in preparing nanoemulsions is described in the literature. For instance, ethyl oleate has been used in nanoemulsions due to its lower molecular size (310.51 g/mol) as related to medium chain triglycerides (~800 g/mol). Furthermore, nanoemulsions formulated with ethyl oleate have demonstrated enhancement in corneal permeation of drugs [25]. Similarly, Tween 80 (up to 10% *w*/*w*) has been used as a surfactant in many commercial ophthalmic preparations which usually do not cause ocular irritation [26]. Due to the longer hydrocarbon chain length of Tween 80, it can broaden the area of the nanoemulsion region [27]. In addition, Soluphor P has demonstrated improved drug penetration across the biological membranes [28].

The pseudoternary phase diagram of the systems consisting of surfactant: Cosurfactant (Tween 80: Soluphor P) mixture (*S*_mix_), oil phase and water was illustrated in Figure 1A. The binodal curve separating two phase and one phase in the pseudoternary phase diagram was indicated by the visual observation of the sample appearance from turbid to transparent or vice versa. To complete the entire nanoemulsion domain, in addition to the water titration method, oil and water components were fixed and surfactant component varied. Similarly, water and *S*_mix_ components were fixed and the oil component varied. The nanoemulsion domain obtained by these trials at ratios of 1:1, 1:2 and 2:1 with surfactant (Tween 80) to cosurfactant (Soluphor P) ratio were plotted in the phase diagram (Figure 1A). The phase behavior study revealed that, when the surfactant to cosurfactant ratio was 1:2, the maximum quantity of oil can be included in the nanoemulsion system. From the phase diagram, it was indicated that the change in phase behavior within the nanoemulsion region is mainly due to the hydrophobic carbon chain length of the oil and the ratio between surfactant and cosurfactant mixture used in the formulation. Therefore, it is likely that the hydrocarbon chain length compatibility among surfactant and oil is an important factor that affects the globule size formation and stability of nanoemulsion as suggested by Schneider et al. [29]. 

### 3.2. Formulation Optimization 

The nanoemulsions selected for the optimization were evaluated for parameters like droplets size, polydispersity index, zeta potential, and conductivity. The droplets size was optimized as a response in simplex design. The droplets size was in the range of 28.78–81.04 nm. Regression summary output, full model confirmed that components A (*S*_mix_), B (oil), C (water), AB and BC significantly affect the response (droplets size). Whereas AC has *p* value >0.05 suggests that it does not significantly affect the response. Therefore, reduced model was developed eliminating AC term and simplex coefficients were obtained from regressions summary output using Microsoft Office Excel^®^ 2007. *R* Square value of 0.999 suggests the suitability of the model and the lower stand error value of 1.9505 suggests minimum error in the model.

**Figure 2:**
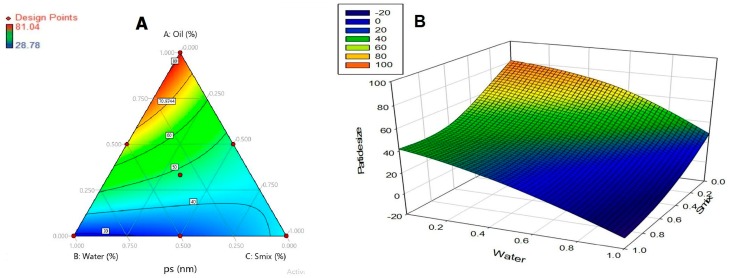
Contour plot (**A**) over the simplex space and response surface graph (**B**) representing nanoemulsion particle size (nm).

Response was calculated over the simplex space, and a contour diagram is plotted (Figure 2). It is inferred from the contour plot that particle size is minimum, when the water component is at higher level (1) and oil and surfactant components are at lower level (0). The optimized formulation of the nanoemulsion consists of ethyl oleate (4%), Tween 80 (12%), Soluphor P (24%) and water (60%). Moxifloxacin was included at a concentration of 0.5% *w*/*w* in all tested formulations.

### 3.3. Validation of Applied Design

The simplex equation of response, droplets size is derived as follows:

*Y* = 40.76 *A* + 81.16 *B* + 27.72 *C* − 55.72 *AB* + 48.71 *BC*

The result at the extra-design checkpoint is predicted based on the equation to confirm the validity of the applied design. It was found that the observed value was close to the practically measured value and hence establishes the effectiveness of the tested mathematical model.

### 3.4. Characterization of Nanoemulsion

Various physicochemical characteristics of prepared nanoemulsions (MM1–MM7) were determined and summarized in Table 2. The drug content of nanoemulsions were in the range of 90–105%. The pH of formulations was ~6–7 and was comparable to commercial ophthalmic drops (pH 6.8), which is likely to be buffered by the lachrymal fluid and may not induce any ocular irritation, reflex tears or rapid blinking of the eye. The developed nanoemulsion shows percentage transmittance value >90% which proves the transparency of the system and droplets are in the nanometer dimensions, confirmed by the average value obtained for droplets size (29–81 nm). The conductivity of nanoemulsions was in the range of 0.08–0.2 mS/cm. No phase separation was observed when diluted 10 times with continuous media in all prepared nanoemulsions. The polydispersity index value of 0–0.5 indicates homogenous, uniformly sized, spherical vesicles. As shown in Table 2, MM1–MM7 have polydispersity indices ranged from 0.24–0.39, which indicate that they were more uniform and homogenous. According to the classical electrical double layer theory, zeta potential value above ±30 mV demonstrates moderate repulsion between similar charged particles, thereby decreasing flocculation or aggregation and potentially stabilizes the dispersion. The observed zeta potential values of MM1–MM7 (0.28–0.38 mV) were comparable, signify the formulation components have not influenced the zeta potential in the current experimental conditions. Viscosity is another important parameter as it can significantly influence the corneal retention time as well as ocular bioavailability from an ophthalmic product. The prepared nanoemulsions have viscosity range from 3.2–6.5 cP, which renders it easily pourable during instillation into the eye and is comparable to normal human tear fluids [30]. Based on the physiochemical properties observed, it seems nanoemulsion MM3 possesses the smallest droplets size and the lowest viscosity as compared to other nanoemulsions prepared. Hence, the nanoemulsion MM3 was selected for further investigation. 

### 3.5. TEM

**Figure 3:**
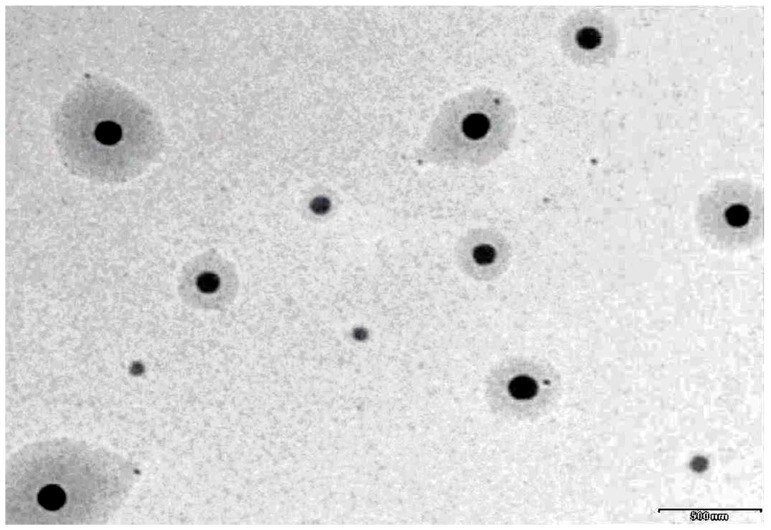
A representative transmission electron microscopy image of moxifloxacin nanoemulsion (MM3).

A representative TEM image of prepared moxifloxacin nanoemulsion (MM3) is shown in Figure 3. It is evident from Figure 3 that the prepared system has droplets in circular shape with uniform size and can be easily distinguished. The droplets appear darker with bright background and are randomly dispersed without any agglomeration throughout the field.

### 3.6. In Vitro Release

**Figure 4:**
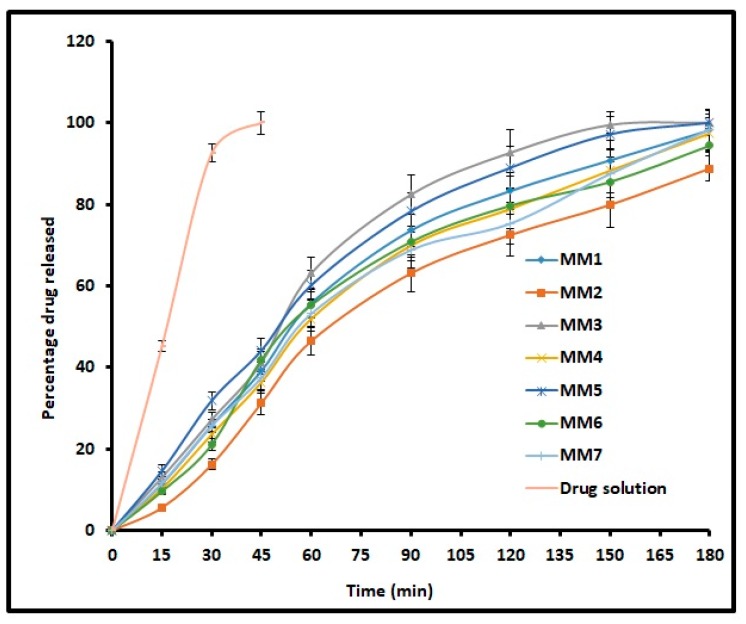
Comparison of percentage moxifloxacin release from prepared nanoemulsions (MM1–MM7) and drug solution (control). The data represents average ± SD of six trials.

The release of drug from nanoemulsions is essential for absorption as well as its therapeutic effect. Figure 4 compares the cumulative percentage of moxifloxacin released at periodic time intervals from MM1–MM7 and control. It is evident from Figure 4 that the drug release profile showed a similar trend, increased steadily over time and is more than 90% in 3 h for all formulations. However, the release of moxifloxacin from control was rapid and complete in 45 min. Amongst all designed o/w nanoemulsion formulations, MM3 showed complete drug release in 150 min. Overall, the release profiles indicate the ability of prepared nanoemulsions to provide a steady drug release for 3 h, which in turn can prolong the therapy. The release mechanism for MM3 was studied using various models and the values are summarized in Table 3. It is evident from the Table 3 that the release kinetics of moxifloxacin from MM3 was fitted into the Higuchi model displaying high *r*^2^ value (0.9486), least SSR value (613.14) and F value (87.59). Thus, the release of moxifloxacin from MM3 was due to Higuchi diffusion controlled mechanism. Further, the n value was less than 0.5 which indicates that drug release in MM3 is mainly by Fickian diffusion. 

### 3.7. Ex Vivo Permeation 

**Figure 5:**
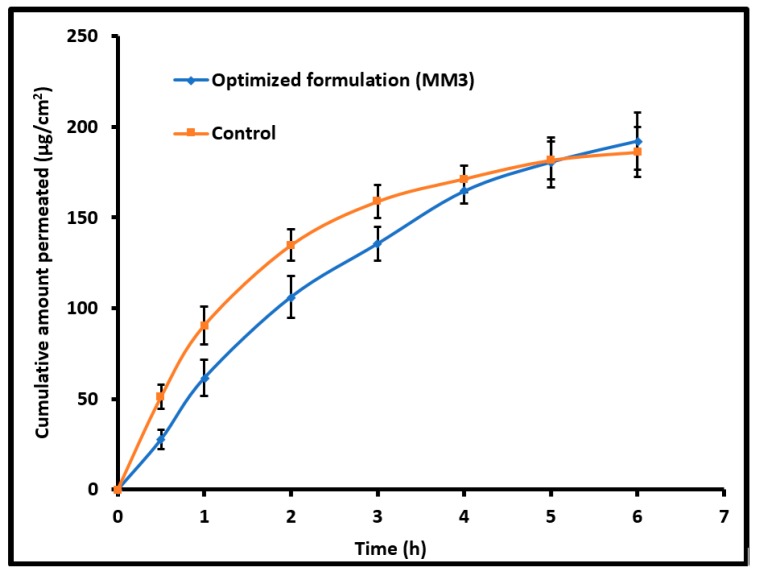
Comparison of moxifloxacin ex vivo permeation across the isolated rabbit cornea membrane from optimized nanoemulsion (MM3) and control (commercial eye drops). The data represents average ± SD of six trials.

The diffusion of therapeutic molecules into and across the biological membrane is mainly influenced by the drug’s physicochemical properties, physiological characteristics of the membrane and different transport routes available for permeation [18]. Figure 5 compares the amount of moxifloxacin transported across the isolated rabbit cornea membrane from MM3 and control (commercial eye drops). A typical permeation profile was exhibited by both MM3 and control and the steady state flux values were comparable (MM3; 32.01 µg/cm^2^/h and control; 31.53 µg/cm^2^/h). The flux value observed here signifies that the physicochemical characteristics of MM3 are suitable for cornea permeation. The permeation rate was relatively high in the first hour with control as compared to MM3. This is probably due to the unique structure of moxifloxacin along with biphasic solubility (both lipophilic and aqueous solubility) and high lipophilicity which would have assisted its easy permeation through the corneal membrane [31].

### 3.8. Antimicrobial Efficacy

It is essential to perform microbiological efficacy studies to demonstrate the activity of the drug in the nanoemulsion against the commonly susceptible microorganisms [32]. The optimized formulation (MM3) showed microbicidal activity, when microbiological testing was performed by the cup plate technique. Visible zones of inhibition were noticed in case of MM3 and control formulation. The diameter of the zone of inhibitions generated by MM3 against both test organisms (*Pseudomonas aeruginosa* and *Staphylococcus aureus*) were either similar or more than that produced by commercial ophthalmic drops (Table 4). The broad-spectrum antibacterial activity of moxifloxacin in the nanoemulsion against the susceptible pathogens was similar to the reference formulation in terms of antimicrobial efficacy. This clearly indicates that the prepared formulation did not change the inherent bactericidal effect of the incorporated moxifloxacin.

### 3.9. Ocular Irritation 

The eye irritation potential of the inducing agent was classified into four grades; practically non-irritating, score 0–3; slightly irritating, score 4–8; moderately irritating, score 9–12; and severely irritating (or corrosive), score 13–16 [20]. The eye irritation score was calculated by dividing the total score for all rabbits by the total number of rabbits tested. The observed eye irritation score in the control was 0.33 and for MM3 was 0.66, which signifies excellent ocular tolerance. Moreover, no ocular damage or abnormal clinical signs pertaining to the cornea, iris, or conjunctivae were visible. In addition, no signs of redness, watering of the eye or swelling were noticed for both MM3 and control. Overall the results of this study revealed that MM3 is safe for ocular application. 

### 3.10. Pharmacokinetics in the Aqueous Humor

**Figure 6:**
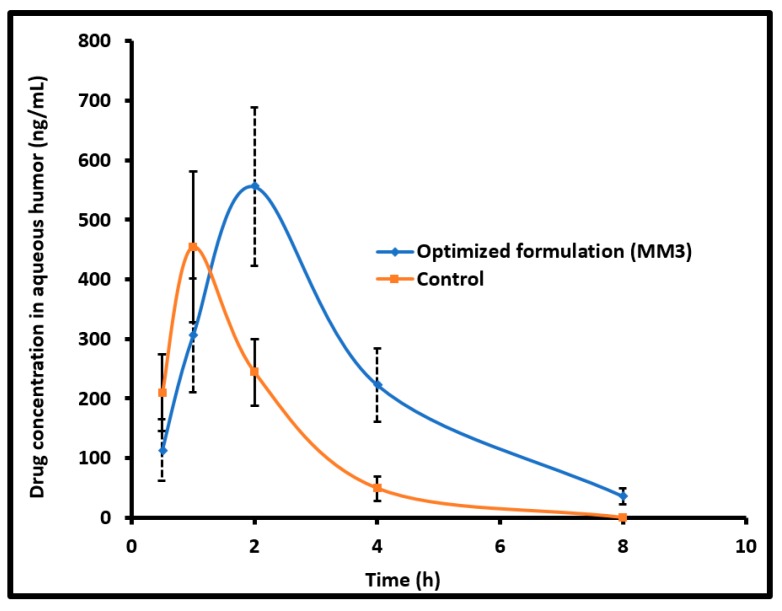
Comparison of mean moxifloxacin concentration in the aqueous humor following topical installation of optimized nanoemulsion (MM3) and control (commercial eye drops) in rabbits. The data represents average ± SD of six trials.

The concentration of the drug permeated into the aqueous humor after administration to the rabbit eyes was quantified to evaluate the ocular bioavailability of moxifloxacin from MM3 as well as the control. Different pharmacokinetic properties were analyzed by using a non-compartmental method [33]. The determined pharmacokinetic parameters are summarized in Table 5. Figure 6 compares the mean moxifloxacin concentration in the aqueous humor following topical installation of MM3 and control in rabbits. It is evident from Figure 6 that the kinetic profiles are distinct for MM3 and control. Indeed, moxifloxacin absorption was rapid and available in the aqueous humor after 30 min in MM3 (113.98 ± 51.45 ng/mL) and control (209.44 ± 64.53 ng/mL), however, the amount of drug was different. At 1 h, the drug level in the aqueous humor increased to 305.99 ± 94.95 ng/mL and 454.19 ± 126.91 ng/mL in MM3 and control, respectively. The drug absorption in MM3 was further prolonged and the drug level in the aqueous humor continued to rise to attain the peak drug concentration (*C*_max_; 555.73 ± 133.34 ng/mL) and the time corresponding to peak concentration (*T*_max_) was 2 h (which is 1 h in control). Comparing the drug absorption with the ex vivo permeation data, one can easily corroborate that the delay in absorption of moxifloxacin into the aqueous humor in MM3 is probably due to its slow permeation rate in the first hour observed in Figure 5. Followed by the rapid absorption, the drug level declined in both groups. At 4 h, the drug level in aqueous humor was considerably higher in MM3 (*p* < 0.0001) as compared to control. At 8 h, the drug level in aqueous humor for MM3 was 35.90 ± 13.01 ng/mL while no moxifloxacin was detected in the control. The mean value of area under the aqueous humor concentration versus time curve (*AUC*_0–8 h_) for MM3 (Table 5) was ~2 fold higher (*p* < 0.001), relative to the control, suggesting significant improvement in ocular bioavailability by MM3 in comparison to the control. The possible reason for the greater observed *AUC* could be due to the longer retention of vesicles (MM3) in the ocular surface which in turn prolongs the contact time of medicament with the eye and thereby improve corneal penetration, when compared to conventional eye drops, as described in the literature [34].

### 3.11. Stability Assessment

Stability studies were conducted to determine the influence of various excipients on drug in MM3 and also to determine physicochemical characteristics of the finished product during storage. After stability testing, the nanoemulsion was found to be a transparent biphasic solution and exhibited no coagulation, aggregation or precipitation. No significant change in pH of MM3 was observed during the stability period. Drug content of MM3 was more than 95% during storage period. There was no phase separation or creaming on visual observation and it was found stable after cooling centrifugation. The data observed indicate minimum changes in stability indicating parameters of nanoemulsion systems like droplets size of MM3 after three months of storage. 

## 4. Conclusions

The nanoemulsion system of moxifloxacin was successfully formulated by developing pseudoternary phase diagram of different proportions of ethyl oleate, Tween 80, Soluphor P and water. It is inferred from the contour plot that particle size is minimum when the water component is at a higher level (1) and oil and surfactant components are at lower level (0). The developed nanoemulsions have optimum viscosity for instillation in eye. They show transmittance value >95% which proves the transparency of the system and the droplets are in nanometer dimensions. The optimized nanoemulsion (MM3) showed adequate antimicrobial effect of the entrapped moxifloxacin and is safe for ocular application. The moxifloxacin level in the aqueous humor was prolonged by MM3 which is greater than the minimum concentration necessary for the therapeutic efficacy (i.e., 0.2 μg/mL or >>>MIC_90_ for aerobic gram-positive and gram-negative pathogens responsible for causing eye infections), signifying the importance of nanoemulsion for ocular therapy. In a nutshell, the optimized nanoemulsion formulation (MM3) could be a promising and viable drug delivery system for effective delivery of moxifloxacin for treatment of various bacterial eye infections. 

## Figures and Tables

**Table 1:** Formulation composition used for preparing nanoemulsions after applying simplex lattice design.

Formulations	Run	Formulation Components			Transformed Proportion		
		*S*_mix_ (%)	Oil (%)	Water (%)	Smix	Oil	Water
MM1	1	52	4	44	1	0	0
MM2	2	36	20	44	0	1	0
MM3	3	36	4	60	0	0	1
MM4	4	44	12	44	0.5	0.5	0
MM5	5	44	4	52	0.5	0	0.5
MM6	6	36	12	52	0	0.5	0.5
MM7	7	41.33	9.33	49.33	0.33	0.33	0.33
MM8 *	8 *	37	18	45	0.063	0.875	0.063

* Check point batch.

**Table 2:** Physicochemical characteristics of prepared nanoemulsions *.

Parameter	MM1	MM2	MM3	MM4	MM5	MM6	MM7	MM8 **
Drug content (%)	96.05 ± 4.01	93.38 ± 2.92	99.90 ± 2.62	94.47 ± 3.48	101.62 ± 2.29	102.06 ± 2.47	98.25 ± 3.78	95.34 ± 4.62
pH	6.33 ± 0.41	6.70 ± 0.52	6.64 ± 0.43	6.85 ± 0.32	6.19 ± 0.28	6.22 ± 0.44	7.04 ± 0.54	6.51 ± 0.36
Transmittance (%)	97.22 ± 5.72	95.38 ± 5.29	97.82 ± 3.83	96.17 ± 3.92	97.71 ± 3.94	95.46 ± 4.42	96.43 ± 4.85	97.62 ± 3.63
Conductivity (mS/cm)	0.11 ± 0.02	0.08 ± 0.01	0.20 ± 0.08	0.14 ± 0.04	0.15 ± 0.07	0.16 ± 0.06	0.14 ± 0.03	0.11 ± 0.03
Dilution potential	>10 times	>10 times	>10 times	>10 times	>10 times	>10 times	>10 times	>10 times
Droplets size (nm)	41.82 ± 13.71	81.04 ± 15.35	28.78 ± 10.34	47.56 ± 16.28	32.41 ± 14.70	67.15 ± 15.84	47.42 ± 14.11	75.99 ± 16.36
Polydispersity index	0.35 ± 0.05	0.26 ± 0.03	0.38 ± 0.06	0.39 ± 0.04	0.34 ± 0.02	0.30 ± 0.05	0.39 ± 0.03	0.24 ± 0.02
Zeta potential (mV)	−0.33 ± 0.01	−0.35 ± 0.02	−0.38 ± 0.012	−0.28 ± 0.02	−0.32 ± 0.03	0.37 ± 0.03	−0.29 ± 0.02	−0.32 ± 0.01
Viscosity (cP)	4.81 ± 1.67	6.50 ± 1.17	3.28 ± 1.42	5.80 ± 1.37	4.57 ± 1.47	5.86 ± 2.44	4.92 ± 1.85	6.39 ± 2.24

* Mean ± SD (*n* = 3); ** Check point batch.

**Table 3:** Model fitting for selected nanoemulsion (MM3).

Model Name	Multiple *R*	*R* Square	*X* Variable	Slope	SSR	Fischer Ratio
Zero order	0.9486	0.8999	0.5862	12.7455	1194.4235	170.6319
First order	0.9558	0.9135	−0.0166	2.3706	20804.4358	2972.0623
Higuchi	0.9740	0.9486	8.7765	−10.4853	613.1492	87.5927
Korsmeyer–Peppas	0.9766	0.9538	0.8446	−1.8055	1086.6082	155.2297
Weibull Model	0.9904	0.9810	1.6232	−2.8693	4932.8215	704.6888
Hixson–Crowell	0.9940	0.9880	0.0271	−0.2801	5019.6483	717.0926

**Table 4:** Antimicrobial efficacy of the optimized formulation (MM3).

Concentration (μg/mL)	Zone of Inhibition in (cm)	
	Control *	MM3
	*Staphylococcus aureus*	
1	1.63 ± 0.22	1.63 ± 0.26
10	2.44 ± 0.31	2.58 ± 0.18
100	3.52 ± 0.25	3.94 ± 0.24
	***Pseudomonas aeruginosa***	
1	1.82 ± 0.34	1.97 ± 0.16
10	2.77 ± 0.23	3.05 ± 0.21
100	4.05 ± 0.25	4.71 ± 0.29

* Control: Commercial eye drops of moxifloxacin.

**Table 5:** Mean pharmacokinetic parameters of moxifloxacin in aqueous humor following topical installation of nanoemulsion (MM3) and control in rabbits.

Parameter	Nanoemulsion (MM3)	Control
*T*_max_ (h)	2	1
*C*_max_ (ng/mL)	555.73 ± 133.34	454.19 ± 126.91
*AUC*_0–8_ (ng.h/mL)	1859.76 ± 424.51 *	958.63 ± 206.84

* Significant difference (*p* < 0.001) observed in moxifloxacin level in nanoemulsion (MM3) group compared to control. Area under the aqueous humor concentration versus time curve (*AUC*_0–8 h_).
